# Evidence that justifies reviewing policies of access to medicines for chronic obstructive pulmonary disease in the Brazilian Public Health System

**DOI:** 10.31744/einstein_journal/2020CE5754

**Published:** 2020-11-12

**Authors:** Charleston Ribeiro Pinto, Lindemberg Assunção Costa, Antônio Carlos Moreira Lemos, Eduardo Martins

**Affiliations:** 1 Universidade Federal da Bahia Faculdade de Farmácia SalvadorBA Brazil Faculdade de Farmácia, Universidade Federal da Bahia, Salvador, BA, Brazil.; 2 Universidade Estadual do Sudoeste da Bahia Curso de Farmácia JequiéBA Brazil Curso de Farmácia, Universidade Estadual do Sudoeste da Bahia, Jequié, BA, Brazil.; 3 Universidade Federal da Bahia Complexo Hospitalar Universitário Professor Edgard Santos SalvadorBA Brazil Complexo Hospitalar Universitário Professor Edgard Santos, Universidade Federal da Bahia, Salvador, BA, Brazil.

Dear Editor,

Szpak et al., demonstrated an expressive increase in expenses by the State Health Authority of Paraná associated to judicialization of access to tiotropium bromide for treatment of chronic obstructive pulmonary disease (COPD), and the relevant individual financial impact provided by the drug.^(^^1^^)^ Based on these findings, the authors indicated the need to revise the clinical protocol of the disease, and insert new therapeutic options on the National Formulary of Essential Medicines. However, although we agree with this observation, we understand the rationale presented by the authors lacks more consistent evidence to guide the process of adding technologies to the Brazilian Public Health System (SUS - *Sistema Único de Saúde* ).

A joint analysis of the official lists of medicines distributed for free (Basic Component of SUS Pharmaceutical Services) and the most recent guidelines of the Global Initiative for Chronic Obstructive Lung Disease (GOLD) shows the therapeutic resources currently available to treat the disease address mainly individuals with less severe cases.^(^^2^^–^^4^^)^ Another limitation identified is overlapping of medicines in both lists, such as short-acting bronchodilators and inhaled corticosteroids (ICS), The latter are not recommended as monotherapy, for being less effective that combined with a long-acting beta2-agonist (LABA), besides increasing the risk of pneumonia in this population.^(^^4^^)^

Moreover, real life evidence suggests many COPD patients are inappropriately treated at SUS.^(^^5^^,^^6^^)^ Julian et al., analyzed the standard treatment of the disease in a national sample of 301,975 patients, in 2017, and found the combined use of ICS/LABA in approximately 92% of patients.^(^^5^^)^ The authors drew attention to overtreatment of the disease for this population, due to excessive use of ICS, which can increase the costs of treatment as well as the risks of adverse events. On the other hand, we cannot rule out the hypothesis of undertreatment of these patients be triggered by less often use of long-acting muscarinic antagonists (LAMA), which are therapeutic resources with robust scientific evidence, which are effective in reducing exacerbations and hospitalization related to the disease.^(^^4^^)^

Considering the context of limited access to medicines to treat the condition, it is worth emphasizing the efforts of management of pharmaceutical services in some Brazilian states to give access to LAMA by means of state protocols. A study carried out by Melo et al., demonstrated the group of states that included tiotropium bromide to treat COPD reduced the number of hospitalizations by 52.4%, as compared to those that did not add the drug to prescription (43.3/100 thousand *versus* 90.0/100 thousand, p<0.01).^(^^7^^)^ This strategy suggests a positive effect in the pharmacoeconomic aspect.

Finally, efforts aiming to assure better conditions to manage COPD are necessary to reduce the social and economic impact of the disease in the health system. Hence, it is fundamental to revise the current policies of access to medicines for this disease.
