# Supplementary material for: Evidence that justifies reviewing policies of access to medicines for chronic obstructive pulmonary disease in the Brazilian Public Health System
Source: Einstein (Sao Paulo). 2020 Nov 12;18:eCE5754. doi: 10.31744/einstein_journal/2020CE5754 (PMC7664825; doi:10.31744/einstein_journal/2020CE5754)
Supplement: Translation

# Translation: Evidências que justificam a revisão das políticas de acesso a medicamentos para doença pulmonar obstrutiva crônica no Sistema Único de Saúde

**Authors:** Charleston Ribeiro Pinto, Lindemberg Assunção Costa, Antônio Carlos Moreira Lemos, Eduardo Martins

**Affiliations:** 1 SalvadorBA Brasil Faculdade de Farmácia, Universidade Federal da Bahia, Salvador, BA, Brasil.; 2 JequiéBA Brasil Curso de Farmácia, Universidade Estadual do Sudoeste da Bahia, Jequié, BA, Brasil.; 3 SalvadorBA Brasil Complexo Hospitalar Universitário Professor Edgard Santos, Universidade Federal da Bahia, Salvador, BA, Brasil.

Caro Editor,

Szpak et al., evidenciaram um aumento expressivo dos gastos da Secretaria Estadual de Saúde do Estado do Paraná com a judicialização do acesso ao brometo de tiotrópio para tratamento da doença pulmonar obstrutiva crônica (DPOC), e relevante impacto financeiro individual proporcionado por esse medicamento.^(^^1^^)^A partir desses achados, os autores apontam para a necessidade de revisão do protocolo clínico da doença e de inserção de novas opções terapêuticas na Relação Nacional de Medicamentos Essenciais. Contudo, apesar de concordarmos com essa observação, entendemos que a racional apresentada pelos autores carece de evidências mais consistentes para nortear o processo de incorporação de tecnologias no Sistema Único de Saúde (SUS).

Uma análise conjunta das listas oficiais de fornecimento gratuito de medicamentos (Componente Básico da Assistência Farmacêutica) e das diretrizes mais recentes da *Global Initiative for Chronic Obstructive Lung Disease* (GOLD) revela que os recursos terapêuticos atualmente disponíveis para tratamento da doença atendem prioritariamente indivíduos com menor gravidade.^(^^2^^–^^4^^)^Outra limitação identificada é a sobreposição de medicamentos de ambas as listas, a exemplo de broncodilatadores de curta ação e corticosteroides inalatórios (CI), sendo esses últimos não recomendados como monoterapia, por serem menos efetivos que combinados a um beta2-agonista de longa ação (LABA), além de aumentar o risco de pneumonia nessa população.^(^^4^^)^

Ademais, evidências de mundo real sugerem que muitos pacientes com DPOC são tratados de forma inadequada no âmbito do SUS.^(^^5^^,^^6^^)^Julian et al., ao analisarem o padrão de tratamento da doença em uma amostra nacional envolvendo 301.975 pacientes no ano de 2017, encontraram o uso de combinação de CI/LABA em cerca de 92% dos pacientes.^(^^5^^)^Os autores chamam atenção para sobretratamento da doença nessa população, devido a uso excessivo de CI, o que pode aumentar tanto os custos do tratamento quanto os riscos de eventos adversos. Por outro lado, não podemos descartar a hipótese de o subtratamento desses pacientes ser motivado pela subutilização de anticolinérgicos de longa ação (LAMA), que são recursos terapêuticos de evidência científica robusta efetivos na redução das exacerbações e hospitalizações relacionadas à doença.^(^^4^^)^

Diante do contexto do acesso limitado a medicamentos para tratamento da doença, vale ressaltar os esforços da gestão da assistência farmacêutica de alguns estados do Brasil em propiciar acesso ao LAMA por meio de protocolos estaduais. Estudo conduzido por Melo et al., demonstrou que o grupo de estados que incluiu o brometo de tiotrópio para tratamento da DPOC reduziu em 52,4% o número de hospitalizações em relação aos estados que não o incorporaram (43,3/100 mil *versus* 90,0/100 mil, p<0,01).^(^^7^^)^Essa estratégia sugere efeito positivo, do ponto de vista farmacoeconômico.

Por fim, esforços visando à garantia de melhores condições para o gerenciamento da DPOC são necessários para reduzir o impacto social e econômico da doença no sistema de saúde. Para tanto, é fundamental a revisão das políticas atuais de acesso a medicamentos para doença.
